# Effect of an exosuit on kinematics in individuals with incomplete spinal cord injury

**DOI:** 10.1177/20556683251375068

**Published:** 2025-10-16

**Authors:** Lara Visch, Brenda E. Groen, Alexander C. H. Geurts, Ilse J. W. van Nes, Noël L. W. Keijsers

**Affiliations:** 1 Department of Research, 6033 Sint Maartenskliniek, Nijmegen, The Netherlands; 2 Department of Sensorimotor Neuroscience, Donders Institute for Brain, Cognition and Behavior, 6034 Radboud University, Nijmegen, The Netherlands; 3 Department of Rehabilitation, Donders Institute for Brain, Cognition and Behavior, 6034 Radboud University Medical Center, Nijmegen, The Netherlands; 4 Department of Rehabilitation, 6033 Sint Maartenskliniek, Nijmegen, The Netherlands

**Keywords:** incomplete spinal cord injury, gait rehabilitation, exosuit, body weight support, kinematics

## Abstract

**Background:**

Anti-gravity exosuits supporting hip and knee extension have emerged for overground training. This study investigated their effect on kinematics in individuals with incomplete spinal cord injury (iSCI) compared to regular walking and to walking with an overground BWS system.

**Methods:**

Fourteen individuals with iSCI were tested during overground walking in three conditions: regular, exosuit, and BWS. Kinematics were assessed using the Xsens MVN motion capture system.

**Results:**

Maximum hip extension was larger for exosuit compared to regular walking (Δ4.7°, 95% CI [1.4, 7.9]), but was not different compared to BWS. Mean knee flexion was smaller for exosuit compared to regular walking (Δ−1.7°, 95% CI [−3.0, −0.4]) and compared to BWS (Δ−3.5°, 95% CI [−5.4, -1.6]). Most secondary outcome measures (e.g. walking speed, stride length, step width, ML COM excursion) showed no differences between exosuit and regular walking. Comparing exosuit to BWS, most secondary outcome measures (e.g., walking speed, stride length, stride time, trunk inclination) favored BWS.

**Conclusions:**

An anti-gravity exosuit resulted in increased hip and knee extension, but did not translate into other gait improvements. Given the more favorable outcomes of the BWS system compared to the exosuit, exosuit design improvements are needed to be effectively implemented in gait rehabilitation after iSCI.

## Introduction

Many individuals with incomplete spinal cord injury (iSCI) experience gait difficulties due to decreased lower extremity strength,^1^ impaired balance,^2^ and sensory impairments.^3^ Therefore, improving gait is a common primary rehabilitation goal in individuals with iSCI.^4^ Traditionally, overground training has been the dominant approach for gait rehabilitation, to facilitate neuromotor recovery.^5^ However, technological advancements have introduced alternative approaches, such as body-weight-supported treadmill training and robotic-assisted training.^3^ These systems ensure safety and facilitate repetitive stepping during gait training.^6–8^ Body weight support (BWS) systems provide a lifting force that reduces the demands on muscle strength and balance control,^9,10^ enabling individuals to start early with gait training while limiting the physical burden on the therapists. While robotic treadmill training is beneficial in the early stages of rehabilitation, overground gait training is more task-specific, which is important for recovery of activities of daily living.^7^

Over the past decade, exosuits have emerged as novel devices for overground gait training.^11^ Exosuits are soft robotic devices without rigid structures that provide additional forces around joints to complement residual mobility and muscle function.^11^ Exosuits that support hip and knee extension during the stance phase of gait, such as the Myosuit,^12^ primarily target the muscles responsible for supporting body weight, commonly referred to as anti-gravity exosuits.^12^ Compared to overground BWS systems, anti-gravity exosuits enable patients to train outdoors and in more everyday home environments,^6^ while BWS systems are expensive and their use is limited to specialized rehabilitation centers.

Gait training for individuals with iSCI should aim to promote walking independence, safety, and improve gait quality while fostering confidence in walking ability.^7^ Both anti-gravity exosuits and BWS systems are viable options for gait training. As they provide support through different mechanisms, their effect on gait patterns may vary due to human-robot interaction.^13^ While the effect of BWS systems on gait patterns have been extensively studied,^14^ research on the effect of anti-gravity exosuits remains limited. In healthy individuals, a natural gait pattern was preserved, with a reduction in knee extensor demand while walking with an anti-gravity exosuit.^15^ In individuals with various gait disorders, an increase in walking speed was observed, however gait kinematics were not investigated.^16^ In a small sample of five adolescents with cerebral palsy larger hip and knee extension have been observed.^17^ In individuals with iSCI, only one case study using an anti-gravity exosuit was performed, which showed increased hip extension without changes in knee angles.^18^ To our knowledge, the effect of an anti-gravity exosuit on gait kinematics and spatiotemporal parameters have not been examined in a larger sample of individuals with iSCI.

The aim of this study was to investigate the effect of an anti-gravity exosuit on kinematics in individuals with iSCI. Kinematics while walking with an anti-gravity exosuit were compared to regular walking without a robotic support system. In addition, we included walking with an overground BWS system as a comparison condition. Reduced hip and knee extension is a gait impairment often seen in individuals with iSCI,^4^ and an anti-gravity exosuit assists by providing additional torques around these joints. We hypothesized that an anti-gravity exosuit would result in increased maximum hip extension and decreased mean knee flexion compared to regular walking without a robotic support system and compared to walking with BWS in individuals with iSCI. Secondary, we expect that this leads to improvements in spatiotemporal gait parameters compared to regular walking without robotic support system. In addition, we expect that the anti-gravity exosuit is a viable alternative to BWS regarding spatiotemporal gait parameters.

## Methods

### Participants

Fourteen individuals with subacute (at least 2 weeks after injury) or chronic iSCI were recruited. Inclusion criteria were: injury level C or D on the American Spinal Injury Association Impairment Scale^19^; walking speed between 0.4 and 0.8 m/s (limited community ambulator)^20^; age ≥ 18 years; reduced hip and/or knee strength indicated by Medical Research Council (MRC) scale scores < 5^21^; and the ability to walk at least 10 m without knee orthoses, walker, or assistance from another person. Participants were excluded if they had: another (neurological) disease that could influence motor performance; skin wounds at exosuit attachment sites; a body height < 150 cm or > 195 cm; or a body weight < 45 kg or > 110 kg. The study was approved by the internal review board of the Sint Maartenskliniek. The Dutch Medical Research Committee of East Netherlands exempted formal ethical approval, because the study was not subject to the Medical Research Involving Human Subjects Act according to Dutch Law (2023-16630). The study was conducted in accordance with the principles of the Declaration of Helsinki (64th WMA General Assembly, Fortaleza, Brazil, October 2013) and the Medical Research Involving Human Subjects Act. Participants signed informed consent before the start of the study.

### Protocol

**Figure 1.**
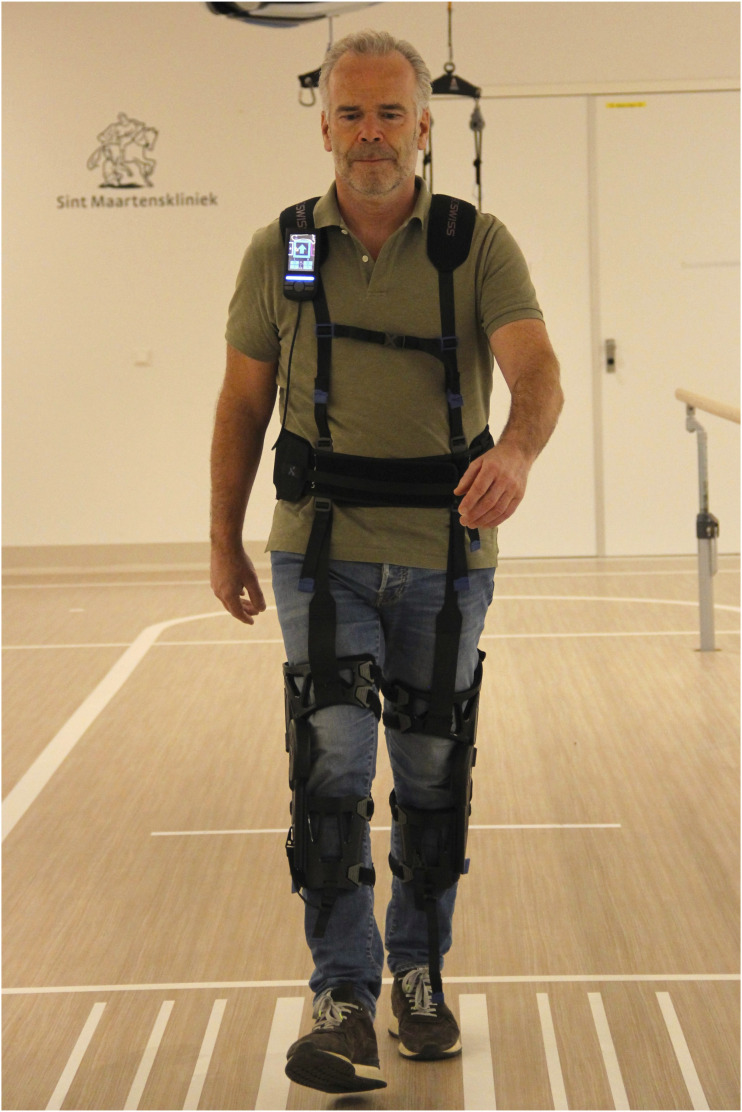
Exosuit (Myosuit).

**Figure 2.**
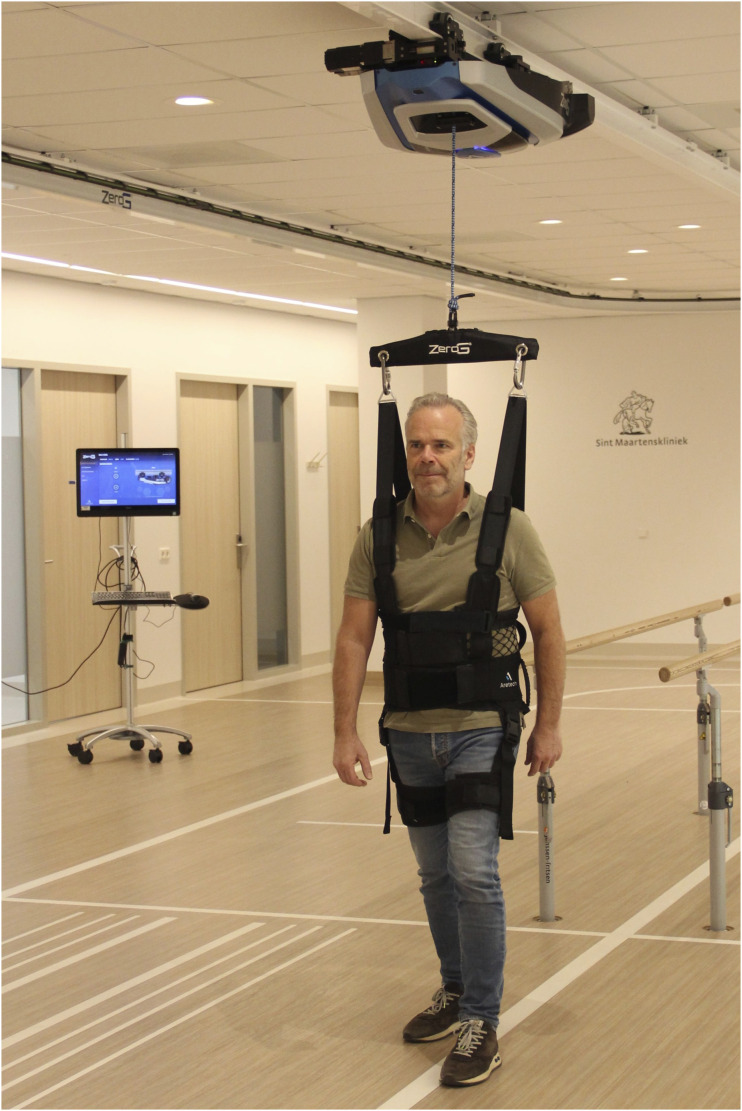
BWS system (ZeroG).

Participants visited the Sint Maartenskliniek for a single measurement session. The anti-gravity exosuit used in this study was the Myosuit (MyoSwiss AG, Zurich, Switzerland) (Figure 1), which weighs 5.5 kg.^16^ It provides active support in hip and knee extension during the stance phase of gait and passive support in hip flexion during the swing phase.^12^ The Myosuit’s assistance levels range from 0 (lowest assistance) to 5 (highest assistance) and can be independently configured for each leg. Participants without prior experience of walking with the Myosuit underwent a half-hour training session before measurements were taken. The BWS system used in this study was the ZeroG (Aretech LLC, Ashburn, Virginia, USA) (Figure 2), a dynamic overground BWS system restricting movement in mediolateral (ML) direction.^22^ The trolley of the BWS system automatically follows the patient during walking, minimizing noticeable horizontal forces in the forward and backward direction. The ZeroG provides BWS support based on a percentage of an individual’s body weight, with a minimum of 4.5 kg. Body weight was measured to provide accurate BWS support. In addition, a fixed level 1 facilitation horizontal force was provided to facilitate gait.^7^ Prior experience with both devices was not controlled in this study. Familiarity with the BWS system was possible, as it is commonly used in subacute gait rehabilitation.

Seventeen inertial measurement units (IMUs) (Xsens MTw Awinda) and the MVN Analyze software (version 2022.0.2) were used to capture kinematic data.^23^ Anatomical measurements, which were used in the MVN Analyze software, and placement of the IMUs were performed in accordance with the manufacturer’s guidelines.^24^ Anatomical measurements included body height (with shoes), shoulder, hip, knee, and ankle height, shoulder and hip width, and elbow, wrist, and arm span. IMUs were placed on the following body segments: the foot, shank, and thigh of each leg, the scapula, upper arm, lower arm, and hand on each side, the sacrum, the sternum, and the head.^25^ The sensors on the shank were placed more distally than prescribed to ensure proper fit of the Myosuit, this position was consistent across conditions. Data were acquired at a frequency of 60 Hz.

Prior to the measurements, a calibration procedure was performed following a standard procedure provided by Xsens.^25^ First, participants stood in the N-pose (fully upright with one foot distance in between both feet) for 4 s. Second, they walked around the room for 15 s. Third, the calibration was processed, and the participants stood still for 8 s.

After the calibration procedure, participants performed overground walking trials in three conditions: regular (walking without a robotic support system), exosuit (walking with the Myosuit), and BWS (walking with the ZeroG). If needed, walking aids (sticks, crutches, or ankle foot orthoses) were permitted and standardized across all conditions. Participants always started with the regular condition, followed by the exosuit and BWS conditions in a random order. The starting condition was fixed. To minimize fatigue, participants were allowed to rest as long as necessary between conditions. For each condition, participants walked back and forth six times over a 12.5 m distance at their preferred walking speed. At the beginning and end of each walking trial, participants stood still for approximately 2 s. Before the exosuit and BWS condition, participants familiarized themselves with the device by walking at least four times back and forth over a 12.5 m distance. The assistance level provided by the Myosuit and BWS was individualized in consultation with a physiotherapist and researcher. For the Myosuit, assistance started at level 1 and was gradually increased to level 4, allowing participants to select the most comfortable assistance level for each leg. Similarly, the level of BWS provided by the ZeroG was individualized for each participant. Participants started with 5% BWS, increasing gradually to 20% BWS in 5% increments, allowing them to choose the most comfortable support level.

### Data analysis

The Xsens MVN Analyze software (version 2022.0.2) was used to process the data, providing an optimal estimate of the position and orientation of each body segment.^25^ Subsequently, the data was exported as mvnx files and analyzed using custom-written scripts in Python (version 3.10 64-bit). The scripts extracted positions of the center of mass (COM), left and right foot segments, sagittal joint angles, and foot contacts from the mvnx files. Positional data were transformed from the global coordinate frame used in the MVN software to a local coordinate frame by a rotation matrix around the z-axis (vertical). The local coordinate frame was defined with its origin at the vertical projection of the COM on the ground at the start of each recording. The x-axis was aligned with the walking direction, determined by the start and end position of the COM projection on the ground for each recording when the participant stood still. The z-axis pointed upwards, and the y-axis completed the right-hand frame. Positions of the COM and sagittal joint angles were time normalized to percent gait cycle (0%–100%) based on initial foot contact of the most affected leg. The most affected leg was determined by asking the participant. The first and last full gait cycle of each recording were excluded from data analysis due to deviations caused by starting and ending in a standing position.

### Outcome measures

The primary outcome measures were maximum hip extension and mean knee flexion of the most affected leg during the stance phase of gait (initial contact - toe-off). Secondary outcome measures were hip and knee range of motion (ROM) of the most affected leg during the stance phase of gait. The following secondary outcome measures were calculated during the entire gait cycle: trunk inclination angle, trunk ROM, and ML COM excursion. The trunk angle was defined as the angle between the sternum sensor and the vertical axis, with the trunk inclination angle calculated as the mean trunk angle over the gait cycle. Hip, knee, and trunk angles were analyzed in the sagittal plane. ML COM excursion was determined as the difference between the maximum and minimum COM values in ML direction. In addition, step width, walking speed, stride length, stride time, and double support phase duration were calculated. Outcome measures were averaged over all gait cycles per condition. Additionally, participant’s experiences were evaluated using a self-customed questionnaire. Participants rated both systems on a scale ranging from 0 to 10 for safety, comfort, ease of walking, confidence, and pain during walking.

### Statistical analysis

For the primary and secondary outcome measures, mean differences and 95% confidence intervals (CI) were calculated for exosuit versus regular walking and exosuit versus BWS.^26^ Differences rather than absolute values were reported, due to the limited accuracy of the absolute values measured by IMUs.^27^ For the primary outcome measures, paired t-tests were conducted using a significance level of 0.025 to account for multiple testing, given the two primary outcome measures (maximum hip extension and mean knee flexion). Effect sizes were calculated using Cohen’s d and interpreted as < 0.2 small effect, 0.2 > or < 0.5 moderate effect, and > 0.5 large effect. Differences in participants’ experiences between both support systems were calculated by subtracting BWS scores from the exosuit scores. Mean differences and 95% CI were calculated. Secondary outcome measures and differences in participants’ experiences were interpreted descriptively via CI. Statistics were performed in R (version 4.2.2).

## Results

### Participants

Fourteen individuals with iSCI (10 males, 4 females) participated in this study, with a mean age of 61 years (standard deviation (SD) = 13) and a mean time since injury of 10 years (SD = 9). Detailed participant characteristics are shown in Table 1. Table 2 provides individual data on preferred walking speed, the assistive device(s) used, and the assistance levels applied in the exosuit and BWS system.

**Table 1.** Participants’ characteristics.

#	Sex	Age (years)	Lesion level	AIS	Cause of injury	TSI (years)	Height (cm)	Weight (kg)	MRC hip extension MAL	MRC knee extension MAL
1	M	59	T4	C	Non-traumatic	8	185	98	1	1
2	M	29	T4	D	Traumatic	8	187	79	Unknown	3
3	M	76	C4	D	Non-traumatic	8	179	76	4	5
4	M	68	C5	D	Traumatic	4	180	84	3	5
5	F	60	T5	D	Non-traumatic	6	173	63	3	4
6	M	66	T4	D	Traumatic	10	180	99	3	3
7	M	57	T12	D	Traumatic	3	191	102	2	5
8	F	80	L3	D	Non-traumatic	16	169	90	4	4
9	M	67	L3	Unknown	Traumatic	23	181	85	0	5
10	M	54	C2	D	Traumatic	34	191	99	4	4
11	M	49	C4	C	Traumatic	7	184	93	2	4
12	F	72	C6	D	Non-traumatic	0.7	168	84	3	5
13	F	69	T9	D	Non-traumatic	0.2	155	60	3	4
14	M	51	C5	D	Traumatic	16.7	184	87	3	4

Abbreviations: M, male; F, female; AIS, American Spinal Injury Association Impairment Scale; TSI, time since injury; MRC, the medical research council scale for muscle strength; MAL, most affected leg.

**Table 2.** Characteristics of the different conditions.

#	Preferred walking speed^a^ (m/s)	Assistive device	Assistance BWS (%)	Assistance exosuit	
				Right	Left
1	0.3	Crutches and AFOs	15	3	3
2	0.6	Stick and AFO	10	2	1
3	0.4	Crutches	15	2	2
4	0.8	None	5	2	2
5	0.7	Crutch	20	1	2
6	0.4	Crutches	15	2	2
7	0.8	Crutch and AFOs	15	2	2
8	0.9	Sticks	10	2	2
9	0.6	Crutches and AFOs	20	2	2
10	0.6	Crutches	15	1	2
11	0.6	Stick	15	2	1
12	0.6	Sticks	15	1	1
13	0.8	Sticks	15	1	1
14	1.0	None	10	2	1

Abbreviations: BWS, body weight support; AFO, ankle foot orthosis.

^a^Preferred walking speed was measured during the regular walking condition.

### Primary outcome measures: Maximum hip extension and mean knee flexion

Primary outcomes measures are shown in Table 3 (upper part). Maximum hip extension was larger for exosuit compared to regular walking (Δ4.7°, 95% CI [1.4, 7.9], t(13) = 3.1, *p* = 0.009, d = 0.8). No significant difference was found in maximum hip extension between exosuit and BWS (Δ3.6°, 95% CI [−0.3, 7.6], t(13) = 2.0, *p* = 0.07). Mean knee flexion was smaller for exosuit compared to regular walking (Δ−1.7°, 95% CI [−3.0, −0.4], t(13) = −2.9, *p* = 0.01, d = −0.2) and smaller compared to BWS (Δ-3.5°, 95% CI [−5.4, −1.6], t(13) = −4.0, *p* = 0.002, d = −1.1).

**Table 3.** Primary and secondary outcome measures.

	Exosuit - Regular		Exosuit - BWS	
	Mean difference	95% CI (difference)	Mean difference	95% CI (difference)
Primary outcome measures				
Maximum hip extension (°)	4.7	[1.4, 7.9]*	3.6	[−0.3, 7.6]
Mean knee flexion (°)	−1.7	[−3.0, −0.4]*	−3.5	[−5.4, −1.6]*
Secondary outcome measures				
Hip ROM (°)	0.3	[−1.3, 1.9]	1.1	[−1.7, 3.9]
Knee ROM (°)	−0.5	[−1.5, 0.4]	−1.2	[−3.3, 0.8]
Trunk inclination angle (°)	1.7	[0.8, 2.6]^a^	1.9	[0.6, 3.3]^a^
Trunk ROM (°)	−0.7	[−1.7, 0.4]	0.9	[0.2, 1.6]^a^
ML COM excursion (cm)	0.6	[−0.03, 1.1]	0.8	[0.09, 1.5]^a^
Step width (cm)	0.1	[−1.5, 1.7]	−4.3	[−8.5, −0.2]^a^
Walking speed (m/s)	−0.04	[−0.09, 0.01]	−0.07	[−0.1, −0.03]^a^
Stride length (m)	−0.04	[−0.08, 0.006]	−0.04	[−0.09, −0.005]^a^
Stride time (s)	0.06	[0.02, 0.1]^a^	0.1	[0.05, 0.2]^a^
Double support phase duration (%)	1.3	[−0.3, 2.9]	2.5	[0.5, 4.4]^a^

Abbreviations: ROM, range of motion; ML, mediolateral; COM, center of mass; BWS, body weight support; CI, confidence interval.

Exosuit - Regular and Exosuit - BWS: a positive value indicates a higher value for the exosuit compared to regular walking or BWS, respectively; a negative value indicates a lower value for the exosuit compared to other conditions.

^a^95% confidence interval does not include zero.

* *p*-value < 0.025.

### Secondary outcome measures

Secondary outcome measures are shown in Table 3 (lower part). Trunk inclination angle and stride time were higher for exosuit compared to regular walking. Hip, knee, and trunk ROM, ML COM excursion, step width, walking speed, stride length, and double support phase duration were not different between exosuit and regular walking. For exosuit compared to BWS, trunk ROM, trunk inclination angle, ML COM excursion, stride time, and double support phase duration were larger and step width, walking speed, and stride length were smaller. Hip and knee ROM were not different between exosuit and BWS.

### Experience of participants

Participants’ experiences with both support systems are shown in Table 4. Higher scores for BWS compared to exosuit were found for safety, comfort, and confidence. Mean differences between exosuit and BWS were: safety −1.3 (95% CI [−2.1, −0.5]), comfort −1.4 (95% CI [−2.4, −0.4]), ease of walking −0.7 (95% CI [−1.4, 0.02]), confidence −0.9 (95% CI [−1.7, −0.05]), and pain 0.3 (95% CI [−0.2, 0.8]).

**Table 4.** Experience of participants.

	BWS	Exosuit
Safety	9.0 ± 1.2	7.7 ± 1.4
Comfort	8.1 ± 1.6	6.7 ± 2.2
Easy	8.5 ± 0.9	7.8 ± 1.4
Confidence	8.4 ± 1.2	7.6 ± 1.2
Pain	0.1 ± 0.5	0.4 ± 0.9

Abbreviations: BWS, body weight support.

Results are presented as means ± standard deviation.

## Discussion

The aim of this study was to investigate the effect of an anti-gravity exosuit on kinematics in individuals with iSCI, and to compare these effects to regular walking without a robotic support system and walking with an overground BWS system. As hypothesized, compared to regular walking, walking with the exosuit increased maximum hip extension and decreased mean knee flexion. When comparing the exosuit to the BWS system, no differences were found in maximum hip extension, whereas mean knee flexion was smaller with the exosuit.

Comparing the exosuit to regular walking, a significant increase in hip extension (4.7°) and knee extension (1.7°) was found. These findings align with a case study involving an individual with iSCI.^18^ A study in five adolescents with cerebral palsy reported larger increases in both hip and knee extension (12° and 6°, respectively), likely due to the pronounced crouch gait in this population.^17^ Although significant increases in hip and knee extension were found in this study, the functional benefits remain uncertain. Biomechanically, the observed increase in hip extension in the current study may enhance gait propulsion and stance stability.^4^ However, no changes were found in walking speed or stride length between conditions. In contrast, a small increase in stride time (Table 3) was observed with the exosuit compared to regular walking. These differences in spatiotemporal parameters align with a previous study in adolescents with cerebral palsy.^17^ Other kinematic or spatiotemporal parameters showed no differences between conditions, except for a small increase in trunk inclination (Table 3). The latter finding reflects a more forward-leaning posture that may be a compensatory strategy to counterbalance the weight of the exosuit’s backpack. The findings indicate that the exosuit used in the current study facilitates hip and knee extension during walking in individuals with iSCI, but that its overall impact on their gait pattern remains limited.

When comparing the exosuit to the BWS system, a near-significant increase in hip extension (3.6°) and a significant increase in knee extension (3.5°) were observed. The increased knee extension aligns with our hypothesis. This can be attributed to the additional torque around the knee joint provided by the exosuit while the BWS system facilitates vertical unloading. Despite the increased knee extension with the exosuit, the BWS system had a more favorable impact on trunk inclination and spatiotemporal parameters. A higher walking speed, longer stride length, and shorter stride time with the BWS system all suggest a greater potential for increasing the number of steps during training, which is an important factor in improving gait in neurological patients.^6,28^ While the mediolateral COM excursion was smaller with the BWS system, step width was unexpectedly larger,^29^ probably due to the harness straps of the BWS system. Although participants rated both support systems as adequate for gait training, the BWS system received higher scores for safety, comfort, and confidence. Despite the increased knee extension with the exosuit, other gait parameters and subjective experiences were more in favorable of the BWS system. Hence, our study suggests that the exosuit may not yet offer advantages over the BWS system in individuals with iSCI. Nevertheless, anti-gravity exosuits can be used to enhance hip and knee extension for training outdoors and in everyday home environments.

Several limitations of this study should be considered. First, we opted for IMUs instead of the gold standard optical motion capture system due to practical reasons and the challenge of marker placement on prescribed anatomical landmarks when using the exosuit or BWS system. Although IMUs may exhibit calibration-related offsets, they are valid for kinematic waveform assessment^30^ and have demonstrated a level of accuracy comparable to that of optical motion capture system for dynamic balance measures.^31^ While this choice limits the accuracy of absolute joint angle measurements, the repeated-measures design supports reliable interpretation of differences between conditions.^27^ Second, participants always performed the regular walking condition first, which could introduce order effects. Third, our findings cannot be directly generalized to a broader population of individuals with iSCI with more severe or minor impairments,^20^ as participants were selected based on the presence of clear gait impairments and their ability to complete the study protocol. Fourth, the current study specifically investigated the Myosuit (exosuit) and ZeroG (BWS system). Due to design differences, our findings may not be applicable to other exosuits and BWS systems. Fifth, the findings are based on a single-session, limiting the insights of long-term use or training effects. Sixth, due to the unknown effects of an anti-gravity exosuit on kinematics, a power analysis could not be conducted, and a convenient sample of limited size was included. Therefore, it was not possible to perform a responder analysis, which could be valuable for future research to identify individuals who may benefit.

In conclusion, an anti-gravity exosuit (Myosuit) resulted in a significant increased hip and knee extension compared to regular walking without a robotic support system in individuals with iSCI. However, these changes did not affect other kinematic or spatiotemporal parameters, suggesting a limited overall impact on their gait pattern. Compared to walking with the BWS system (ZeroG), the exosuit increased knee extension, but the BWS system appeared to be more beneficial regarding other gait parameters and subjective experiences. Hence, the current exosuit needs design improvements to have greater impact on gait to be effectively implemented in gait rehabilitation after iSCI.

## Data Availability

The datasets used during the current study are available from the corresponding author on reasonable request.*

## Appendix

### Abbreviations

AIS: American Spinal Injury Association Impairment Scale
BWS: Body weight support
CI: Confidence interval
COM: Center of mass
IMUs: Inertial measurement units
iSCI: Incomplete spinal cord injury
ML: Mediolateral
MRC: Medical Research Council
ROM: Range of motion
SD: Standard deviation
